# Calibration and discrimination ability of the Dat'AIDS score in people living with HIV aged 70 years and older from the Dat'AIDS cohort

**DOI:** 10.1111/hiv.70207

**Published:** 2026-02-11

**Authors:** Abeo Mousse, Clotilde Allavena, Amandine Gagneux‐Brunon, François Raffi, Laurent Hocqueloux, Claire Genet, Madeline Pascard, Damien Jolly, Firouzé Bani‐Sadr, Lukshe Kanagaratnam, Maxime Hentzien, and The Dat'AIDS Study Group, C. Chirouze, C. Drobacheff‐Thiébaut, A. Foltzer, K. Bouiller, L. Hustache‐Mathieu, Q. Lepiller, F. Bozon, O Babre, AS. Brunel, P. Muret, E. Chevalier, C. Jacomet, H. Laurichesse, O. Lesens, M. Vidal, N. Mrozek, C. Aumeran, O. Baud, V. Corbin, E. Goncalvez, A Mirand, A brebion, C Henquell, I. Lamaury, I. Fabre, E. Curlier, R. Ouissa, C. Herrmann‐Storck, B. Tressieres, MC. Receveur, F. Boulard, C. Daniel, C. Clavel, PM. Roger, S. Markowicz, N. Chellum Rungen, D. Merrien, P. Perré, T. Guimard, O. Bollangier, S. Leautez, M. Morrier, L. Laine, D. Boucher, P. Point, L. Cotte, F. Ader, A. Becker, A. Boibieux, C. Brochier, F Brunel‐Dalmas, O. Cannesson, P. Chiarello, C. Chidiac, S. Degroodt, T. Ferry, M. Godinot, J.M. Livrozet, D. Makhloufi, P. Miailhes, T. Perpoint, M. Perry, C. Pouderoux, S. Roux, C. Triffault‐Fillit, F. Valour, C. Charre, V. Icard, J.C. Tardy, M.A. Trabaud, I. Ravaux, A. Ménard, AY. Belkhir, P. Colson, C. Dhiver, A. Madrid, M. Martin‐Degioanni, L. Meddeb, M. Mokhtari, A. Motte, A. Raoux, C. Toméi, H. Tissot‐Dupont, I. Poizot‐Martin, S. Brégigeon, O. Zaegel‐Faucher, V. Obry‐Roguet, H Laroche, M. Orticoni, M.J. Soavi, E. Ressiot, M.J. Ducassou, I. Jaquet, S. Galie, H. Colson, A.S. Ritleng, A. Ivanova, C. Debreux, C. Lions, T Rojas‐Rojas, A. Cabié, S. Abel, J. Bavay, B. Bigeard, O. Cabras, L. Cuzin, R. Dupin de Majoubert, L. Fagour, K. Guitteaud, A. Marquise, F. Najioullah, S. Pierre‐François, J. Pasquier, P. Richard, K. Rome, JM Turmel, C. Varache, N. Atoui, M. Bistoquet, E Delaporte, V. Le Moing, A. Makinson, N. Meftah, C. Merle de Boever, B. Montes, A. Montoya Ferrer, E. Tuaillon, J. Reynes, B. Lefèvre, E. Jeanmaire, S. Hénard, E. Frentiu, A. Charmillon, A. Legoff, N. Tissot, M. André, L. Boyer, MP. Bouillon, M. Delestan, F. Goehringer, S. Bevilacqua, C. Rabaud, T. May, F. Raffi, C. Allavena, E. Billaud, C. Biron, B. Bonnet, S. Bouchez, D. Boutoille, C. Brunet‐Cartier, C. Deschanvres, B.J. Gaborit, M. Grégoire, O. Grossi, T. Jovelin, M. Lefebvre, P. Le Turnier, R. Lecomte, P. Morineau, V. Reliquet, S. Sécher, M. Cavellec, E. Paredes, A. Soria, V. Ferré, E. André‐Garnier, A. Rodallec, P. Pugliese, S. Breaud, C. Ceppi, D. Chirio, E. Cua, P. Dellamonica, E. Demonchy, A. De Monte, J. Durant, C. Etienne, S. Ferrando, R. Garraffo, C. Michelangeli, V. Mondain, A. Naqvi, N. Oran, I. Perbost, M. Carles, C. Klotz, A. Maka, C. Pradier, B. Prouvost‐Keller, K. Risso, V. Rio, E. Rosenthal, I. Touitou, S. Wehrlen‐Pugliese, G. Zouzou, L. Hocqueloux, C.Mille. T. Prazuck, C. Gubavu, A. Sève, G. Béraud, V. Legros, V. Avettand‐Fènoël, A. Cheret, C. Goujard, Y. Quertainmont, E. Teicher, N. Lerolle, S. Jaureguiberry, R. Colarino, O. Deradji, A. Castro, A. Barrail‐Tran, Y. Yazdanpanah, R. Landman, V. Joly, J. Ghosn, C. Rioux, S. Lariven, A. Gervais, FX. Lescure, S. Matheron, F. Louni, Z. Julia, S. Le GAC C. Charpentier, D. Descamps, G. Peytavin, C. Duvivier, C. Aguilar, F. Alby‐Laurent, K. Amazzough, G. Benabdelmoumen, P. Bossi, G. Cessot, C. Charlier, P.H. Consigny, K. Jidar, E. Lafont, F. Lanternier, J. Leporrier, O. Lortholary, C. Louisin, J. Lourenco, P. Parize, B. Pilmis, C. Rouzaud, F. Touam, MA. Valantin, R. Tubiana, R. Agher, S. Seang, L. Schneider, R. PaLich, C. Blanc, C. Katlama, F. Bani‐Sadr, M. Hentzien, A. Brunet, D. Lambert, C. Strady, M. Moutel, M. Petithomme‐Nanrocki, V. Greigert, I. Kmiec, H. Marty, V. Brodard, C. Arvieux, P. Tattevin, M. Revest, F. Souala, M. Baldeyrou, S. Patrat‐Delon, J.M. Chapplain, F. Benezit, M. Dupont, M. Poinot, A. Maillard, C. Pronier, F. Lemaitre, C. Morlat, M. Poisson‐Vannier, T. Jovelin, JP. Sinteff, A. Gagneux‐Brunon, E. Botelho‐Nevers, A. Frésard, V. Ronat, F. Lucht, D. Rey, P. Fischer, M. Partisani, C. Cheneau, C. Mélounou, C. Bernard‐Henry, E. de Mautort, S. Fafi‐Kremer, P. Delobel, M. Alvarez, N. Biezunski, A. Debard, C. Delpierre, G. Gaube, P. Lansalot, L. Lelièvre, M. Marcel, G. Martin‐Blondel, M. Piffaut, L. Porte, K. Saune, O. Robineau, F. Ajana, E. Aïssi, I. Alcaraz, E. Alidjinou, V. Baclet, L. Bocket, A. Boucher, M. Digumber, T. Huleux, B. Lafon‐Desmurs, A. Meybeck, M. Pradier, M. Tetart, P. Thill, N. Viget, M. Valette

**Affiliations:** ^1^ Department of Internal Medicine, Clinical Immunology and Infectious Diseases Reims University Hospital Reims France; ^2^ UR 3797 “Vieillissement, Fragilité,” Faculty of Medicine University of Reims Champagne‐Ardenne Reims France; ^3^ Department of Research and Public Health Robert Debré Hospital, Reims University Hospitals Reims France; ^4^ Department of Infectious Diseases Nantes Université, CHU Nantes, INSERM Nantes France; ^5^ Infectious Disease Department University Hospital of Saint Etienne Saint‐Étienne France; ^6^ Infectious Diseases Department Orléans University Hospital Orléans France; ^7^ Infectious Diseases Department Limoges University Hospital Limoges France

**Keywords:** aged, cohort, DAT'AIDS, HIV infection, validation study

## Abstract

**Objective:**

The Dat'AIDS score was developed to predict 5‐year mortality risk in people living with HIV aged 60 and older. However, its validity in people living with HIV aged 70 years and older needed confirmation.

**Methods:**

This was a multicentre prospective cohort study in the Dat'AIDS French cohort. We calculated the Dat'AIDS score and Veterans Aging Cohort Study (VACS) indices 1.0 and 2.0 in people living with HIV aged 70 or older, at their first medical visit between 01/06/2014 and 31/12/2017. Participants were followed until 31 December 2019 (before the COVID‐19 era). Discrimination and calibration of the Dat'AIDS score were assessed using Harrell's C‐statistic and comparisons of predicted versus observed survival probabilities. The comparison of the discriminative capacity of the Dat'AIDS score with the VACS indices was performed.

**Results:**

A total of 1330 participants (75.5% male, median age: 73.7 years, median time since HIV diagnosis: 21.7 years, median time under combination antiretroviral therapy (cART): 19.9 years, median CD4 cell count: 553 cells/μL, HIV‐1 RNA ≤50 copies/mL: 88.7%) were included. Overall, 221 (16.6%) deaths were recorded during 5598 patient‐years of follow‐up. The Dat'AIDS score showed good discrimination (C‐statistic: 0.72; 95% confidence interval [CI; 0.68–0.75]). Calibration was good except for the moderate‐risk group (5% difference). The Dat'AIDS score showed better discrimination than VACS 1.0 and 2.0 with albumin, aspartate aminotransferase (AST) and alanine transaminase (ALT) normal value imputation (C‐statistic: 0.72 vs. 0.69 for both) and was similar to VACS 2.0 without imputation (0.72 vs. 0.71), that could be calculated in 99.1%, 98.6% and 34.0%, respectively.

**Conclusions:**

The Dat'AIDS score showed good discrimination and calibration in people living with HIV aged 70 years and older, providing an easy and valuable tool for clinical decision‐making and research.

## INTRODUCTION

Since the advent of combination antiretroviral therapy (cART), both AIDS‐ and non‐AIDS‐related mortality have decreased, and HIV infection has become a long‐term chronic disease [1, 2]. Therefore, there is a new and growing older population with comorbidities among people living with HIV. A recent modelling study in Europe predicted that the proportion of people living with HIV aged 60 years or older will increase from 8% in 2010 to 39% in 2030 [3]. Despite a marked increase in life expectancy, mortality rates among people living with HIV remain 3 to 15 times higher than those observed in the general population [4, 5]. Age‐related comorbidities are significantly more common among people living with HIV, including in younger age groups, compared to the general population of the same age [6]. In France, cardiovascular diseases are the third leading cause of death among people living with HIV [7]. Cancers are a common comorbidity among people living with HIV and accounted for the primary cause of death for 34% of people living with HIV in France in 2010 [2]. The increased risk of cancer‐related mortality was attributed to non‐HIV‐related cancers [8].

However, this growing population has been insufficiently studied, and the identification of clinical scores, incorporating more than classical factors such as CD4 count, has become a research priority to stratify patients at high risk of mortality [9, 10].

For this purpose, the Dat'AIDS score was derived and internally validated in 2018 in the Dat'AIDS cohort [11, 12]. The Dat'AIDS score comprises simple, easily accessible and reliable predictors, that is, HIV‐related factors such as CD4 cell count, and non‐HIV‐associated factors such as age and comorbidities (Table S1). Of note, candidate variables for the score such as HIV‐1 RNA >50 copies/ml, AIDS diagnosis or CD4 nadir were not found to be significant predictors and were not included in the Dat'AIDS score, highlighting the predominant burden of comorbidities in this population. The Dat'AIDS score theoretically ranges from 0 to 73, although observed scores in the derivation study ranged from 0 to 45. The score identifies four risk groups ranging from low to very high risk, with the low‐risk group and the very‐high‐risk group having an expected probability of 5‐year mortality of 5% and 46%, respectively. The score has previously shown good discrimination (C‐statistic, 0.76 [95% confidence interval (CI) 0.71–0.79]) and good calibration in internal validation [11].

The ability of the Dat'AIDS score to predict overall 5‐year mortality in people living with HIV aged 60 or older from 2015 to 2020 was successfully externally validated using data from the Swiss HIV Cohort Study (SHCS). Again, the score demonstrated good discrimination (C‐statistic, 0.73 [95% CI 0.69–0.77]) and good calibration [12].

To date, two prognostic indices have been used specifically in people living with HIV, namely, the Veterans Aging Cohort Study (VACS) index versions 1.0 [13] and 2.0 [14]. The VACS index 1.0 [13] includes organ injury through the Fibrosis‐4 Index for Liver Fibrosis (Fib‐4 index), estimated glomerular filtration rate (eGFR) and HIV‐related factors such as viral load and CD4 cell count. It has recently been updated, and version 2.0 [14] adds, among other variables, body mass index (BMI), platelet count, white blood cell count and albumin level.

The derivation cohort for the Dat'AIDS score included fewer than 200 people living with HIV aged 70 and older (approximately 15%), and while there were 341 (also around 15%) people living with HIV aged 70 or over in the Swiss HIV cohort used for external validation [11, 12]. Therefore, it is of critical importance to assess the validity of the score in this subgroup, which will continue to grow in number in the coming years. We aimed to evaluate the calibration and discriminative capacity of the Dat'AIDS score among people living with HIV aged 70 years and older. Additionally, we compared the discriminative capacity of the Dat'AIDS score to that of the VACS index 1.0 and 2.0 in this population.

## MATERIALS AND METHODS

### Study design and population

This study recruited persons from the Dat'AIDS French national multicentre cohort. Dat'AIDS is a prospective French multicenter cohort of people living with HIV established in 2000. As of 2018, it comprised 24 French public hospitals, including French overseas territories (the islands of Martinique and Guadeloupe in the Caribbean). It covers approximately half of the people living with HIV in care in France, with more than 90 000 patients currently included [12].

It is based on a computerized real‐time medical record that is used by clinicians who collect demographic, behavioural, epidemiological, clinical and biological data in a dedicated database during consultations, using coded identification numbers. All study participants were orally informed and had access to information about the study on the Dat'AIDS website (www.dataids.org). They had the possibility to explicitly oppose the use of their medical records data for research purposes. The Dat'AIDS cohort is registered on ClinicalTrials.gov under the identifier NCT02898987 [15].

To be included in this study, subjects had to be aged 70 years or older, be infected with HIV‐1 and have had at least one visit at their HIV clinic between 1st June 2014 and 31 December 2017. The first medical visit aged 70 or over during this period was taken as the entry date into this study, and the start of follow‐up. The end of follow‐up was determined by the following criteria: the date of the patient's death, completion of 5 years of follow‐up, 31 December 2019 (prior to the COVID‐19 period), whichever occurred first, or else the date of the last recorded follow‐up before 31 December 2019.

For patients lost to follow‐up before the exit date, vital status was retrieved in available medical records to check whether more recent data were available. Subsequently, for patients still lost to follow‐up as of the end of the study period, the centres where they received medical care conducted a comprehensive manual search for their vital status in the French Deceased Individuals Database (https://deces.matchid.io/search?advanced=true&fuzzy=false). If, despite these efforts, no more recent news could be found for a patient, they were then considered to be lost to follow‐up by the medical care centre and were censored at the last date at which they were known to be alive.

Patients were not included in the study if they had HIV‐2 infection or if they did not have at least one CD4 cell count available within the 12‐month period before or after the inclusion date.

### Data collection

We collected the items from the three scores (Dat'AIDS, VACS index 1.0 and 2.0) (Supplementary Table 2) at baseline: age; date of HIV diagnosis; sex assigned at birth, height (meters); weight (kg); country of birth; main HIV acquisition risk group; ongoing antiretroviral therapy (ART) regimen; ART duration; CD4 cell count and CD4 cell count nadir (cells/mm^3^); HIV‐1 RNA (copies/mL); AIDS status; serum creatinine level (μmol/L); haemoglobin (g/dL); history of non‐HIV cancer; cardiovascular disease (CVD); Hepatitis C virus (HCV) co‐infection; active Hepatitis B virus (HBV) co‐infection; cirrhosis; aspartate aminotransferase (AST, U/L); alanine transaminase (ALT, U/L); fibrosis‐4 Index for Liver Fibrosis (Fib‐4 index); albumin (g/L); platelet count (10^3^/μL) and white blood cell count (10^3^ cells/μL).

CVD included history of myocardial infarction, congestive heart failure and cerebrovascular disease [11]. eGFR (mL/min/1.73m^2^) was calculated at baseline using the CKD‐EPI formula [16] and BMI was calculated at baseline as weight (kg)/height^2^ (m^2^). Low BMI and anaemia were, respectively, defined as BMI < 18.5 kg/m^2^ and haemoglobin <13 g/dL for males or <12 g/dL for females. HBV co‐infection was defined as at least one positive hepatitis B surface antigen within 12 months before or after the entry date. HCV co‐infection was defined as at least one positive anti‐HCV antibody (recording the value from the nearest assessment within a time window of up to 3 months after the baseline date). Where appropriate, adapted ICD‐10 coding algorithms for Charlson comorbidity index were used [17, 18].

As the majority of patients in the Dat'AIDS cohort have a stable health status and follow‐up every 6 months (and possible up to every 12 months), with score parameters showing minimal variation during our study period, we considered variables collected within 12 months before and after the entry date (except for BMI to avoid exclusion of the most stable patients who are less likely to have multiple body weight measurements) and selected the values recorded closest to the date of entry. We also preferentially included variables obtained after the entry date, instead of values before, as they were sometimes taken much closer to the patient's first visit, better reflecting the values of the various variables at the entry date. Sensitivity analyses were conducted for this purpose to assess the robustness of our results.

### Statistical analysis

We used median and interquartile range (IQR) and number (percentage) to describe population characteristics. As the three scores (Dat'AIDS, VACS index 1.0 and 2.0) were designed for prognostic purposes, these were only calculated once at baseline, and not in a time‐dependent manner.

Patients were ranked according to the four risk levels (low, moderate, high, very high) defined by the Dat'AIDS score. Observed survival probabilities were obtained for each of the four groups and plotted using Kaplan–Meier curves.

We evaluated the discrimination capacity of the Dat'AIDS score in continuous using Harrell's C‐statistic [16] and hazard ratios (HRs) between predefined risk groups in a Cox proportional hazards regression model. In addition, the C‐statistic was evaluated in the following subgroups: gender; CD4 count (<350 or ≥350 cells/μL); CD4 nadir (<200 or ≥200 cells/μL); CD4 count (< or ≥median) and plasma HIV‐1 RNA (<200 or ≥200 copies/mL). A C‐statistic <0.6 was considered as being of no clinical value, 0.6–0.7 of some clinical value, 0.7–0.8 as good discrimination and >0.8 as an indication of excellent discrimination [19, 20, 21].

Calibration of the Dat'AIDS score in continuous was evaluated comparing predicted [11] versus observed (Kaplan–Meier) survival probabilities using the calibration intercept and slope computed on the Poisson regression representation of a Cox model [22, 23]. Finally, we assessed agreement between predicted [11] and observed (Kaplan–Meier) survival probabilities for each predefined risk group visually. Predicted probabilities were calculated using the baseline survival function of the derivation study as previously published [11].

According to preliminary analyses, we observed more deaths within our population than were observed in the derivation dataset or in the external validation study [11, 12]. Therefore, it was anticipated that our study would have sufficient power to assess calibration and discrimination. Moreover, it has been demonstrated that, for external validation of a prognostic model, a minimum of 100 events is required, and ideally 200 (or more) events [24].

To compare the Dat'AIDS score with the VACS 1.0 and 2.0 indices, we assessed their discrimination capacity using the C‐statistic. Each comparison was conducted among patients for whom we could simultaneously calculate both scores. We chose to compare the Dat'AIDS and VACS 2.0 scores calculated by imputing the BMI in the same way (using the BMI value closest to the inclusion date without any time limitations) to reduce missing data. We also assessed the VACS 2.0 index with BMI imputed similarly, but also setting missing values to normal when albumin (4 g/dL) or one of ALT (25 IU/L) or AST (20 IU/L) was missing, as previously done [25].

To assess the robustness of our results, a paired Student *t*‐test was performed to compare the CD4 count in patients before and after their inclusion in the study, to ensure that the variation in CD4 count within the study population was minimal. Additionally, we compared the C‐statistic and calibration intercept and slope of the Dat'AIDS score by calculating it first using the BMI value collected within the 12 months before or after inclusion, and secondly using the BMI value closest to the inclusion date without any time limitations. We also compared the discriminative abilities (C‐statistics) of the VACS 2.0 score, calculated with and without imputation of BMI and albumin, AST and ALT imputation, as described above.

Analyses were performed using R Core Team (2023). R: A language and environment for statistical computing. R Foundation for Statistical Computing, Vienna, Austria (https://www.R-project.org/), with the survival [26] and tidyverse [27] packages. The Dat'AIDS score was computed using the hivscore R package (https://github.com/jaromilfrossard/). Results are reported according to the TRIPOD checklist for prediction model validation [28].

## RESULTS

### Demographic and clinical characteristics

Among 1446 people living with HIV aged 70 years or older followed in the Dat'AIDS cohort during the study period, 1330 (92.0%) were included in the study (Figure S1).

The baseline characteristics of the study population are shown in Table 1. Median age was 73.7 years (IQR [71.6–77.5]). Median age within each of the four increasing risk groups (low, moderate, high, very high) of the Dat'AIDS score was 71.8, 74.1, 77.2 and 77.0 years, respectively. Most patients included were males (1005 (75.5%)), 69.8% were born in France and 6.2% were born in sub‐Saharan Africa. The median number of years since HIV diagnosis was 21.7 [15.0–26.9]. Patients were receiving cART for a median of 19.9 [13.2–23.5] years. Overall, they were immunologically and virologically well controlled, with a median CD4 cell count of 553 [392–726], while 1176 (88.7%) had an HIV‐1 RNA ≤50 copies/mL.

**TABLE 1:** Characteristics of included people living with HIV aged 70 years and older (*n* = 1330).

Participants' characteristics	*N* or median^a^	(%) or [interquartile range]
Sex at birth		
Female	322	(24.2)
Male	1008	(75.8)
Age (years)	73.7	[71.6–77.5]
Country of birth		
France	929	69.8
Sub‐Saharan Africa	82	6.2
Other	245	18.4
Unknown	74	5.6
Time since HIV infection diagnosis (years)	21.7	[15.0–26.9]
HIV acquisition risk group		
Homo & bisexual contact	487	(36.6)
Heterosexual contact	678	(51.0)
Intravenous drug use	3	(0.2)
Other	67	(5.0)
Unknown/inconclusive	95	(7.1)
HIV‐1 RNA		
≤ 50 copies/mL	1176	(88.4)
> 50 copies/mL	150	(11.3)
HIV‐1 RNA in patients with an HIV‐1 RNA >50 copies/mL (copies/mL)	1556	[109–59 580]
Unknown	4	(0.3)
CD4 cell count (cells/mm^3^)	553.0	[392.0–726.0]
CD4 cell count nadir (cells/mm^3^)	174.0	[77.2–275]
Duration of ART exposure (years)	19.9	[13.2–23.5]
On ART	1273	95.7
Current antiretroviral regimen		
2 NRTI + 1 INI	149	(11.2)
2 NRTI + 1 NNRTI	436	(32.8)
2 NRTI + 1 PI	291	(21.9)
Dual therapy	143	(10.7)
Other	254	(19.1)
Unknown	57	(4.3)
HBV co‐infection	17	(1.3)
HCV co‐infection	63	(4.7)
Other variables included in the Dat'AIDS score and VACS indices		
Age (years)		
70–74	809	(60.8)
75–84	486	(36.5)
≥ 85	35	(2.6)
CD4 cell count (cells/mm^3^)		
< 200	77	(5.8)
200–350	197	(14.8)
350–500	252	(18.9)
> 500	804	(60.5)
Non‐HIV‐related cancer	149	(11.2)
Cardiovascular disease^b^	177	(13.3)
eGFR^c^		
< 30 mL/min/1.73 m^2^	47	(3.5)
30–59 mL/min/1.73 m^2^	399	(30.0)
≥ 60 mL/min/1.73 m^2^	884	(66.5)
Cirrhosis	40	(3.0)
BMI^d^ (complete data = 68.3%)	24.2	[21.7–26.6]
BMI^e^ (complete data = 100.0%)	24.2	[21.6–26.6]
Low BMI^f^	72	(5.4)
Anemia^g^	286	(21.5)
Fibrosis‐4 Index (complete data = 99.4%)	1.9	[1.48–2.54]
Alanine aminotransferase (complete data = 99.5%)	23	[17.0–31.1]
Aspartate aminotransferase (complete data = 99.5%)	25	[20.0–31.0]
Platelet count (complete data = 99.8%)	200	[167.0–242.0]
White blood Cell (complete data = 99.2%)	6	[5.0–7.2]
Albumin (complete data = 34.1%)	42	[38–44.4]

Abbreviations: ART, antiretroviral therapy; BMI, body mass index; eGFR, estimated glomerular filtration rate; INI, integrase inhibitors; NRTI, nucleoside reverse transcriptase inhibitors; NNRTI, non‐nucleoside reverse transcriptase inhibitors; PI, protease inhibitor.

^a^ *n* (%) for qualitative variables; median (IQR) for quantitative variables.

^b^ Cardiovascular disease defined as history of myocardial infarction, congestive heart failure or cerebrovascular disease.

^c^ eGFR calculated using the CKD‐EPI formula.

^d^ BMI collected within 12 months before or after the entry date, using the closest value from this date.

^e^ BMI value closest to the inclusion date without any time limitations.

^f^ Low BMI was defined as BMI <18.5 kg/m^2^.

^g^ Anaemia was defined as a haemoglobin level <12 g/dL for females and <13 g/dL for males.

Among the study population, 221 deaths (15.3%) were recorded during a follow‐up period totalling 5598 patient‐years (PY), corresponding to a death rate of 3.95/100 PY. Up to the end of the study period, 849 people living with HIV (63.8%) had complete follow‐up, and 146 (11%) patients were lost to follow‐up, and 114 (8.6%) had a follow‐up duration <5 years.

The median observed Dat'AIDS score was 8 [1–14] (Table 2). The distribution of pre‐specified risk groups, as well as expected survival probabilities and observed 5‐year mortality (using Kaplan–Meier estimate) are presented in Table 3. A total of 340 (25.6%) people living with HIV were in the low‐risk group, 592 (44.5%) in the moderate‐risk group, 193 (14.5%) in the high‐risk group and 205 (15.4%) in the very‐high‐risk group. Observed survival probabilities are presented in Table 3. Supplementary Figure 2 displays the Kaplan–Meier curves of observed 5‐year survival probabilities across Dat'AIDS risk groups.

**TABLE 2:** Dat'AIDS score and VACS 1.0 and 2.0 indices in the study population of people living with HIV aged 70 years and older (*n* = 1330).

Participants' characteristics	Score calculated using the BMI value collected within 12 months before or after the entry date, selecting the value closest to entry date		Score calculated using the BMI value closest to the inclusion date without any time limitations	
	*N* (%)	Median [IQR]	*N* (%)	Median [IQR]
Dat'AIDS score	908 (68.3)	8.0 [1.0–14.0]	1330 (100.0)	8.0 [1.0–14.0]
VACS index 1.0	1318 (99.1)	43.0 [37.0–55.0]	1318 (99.1)	43.0 [37.0–55.0]
VACS index 2.0	294 (22.1)	66.1 [58.8–74.6]	452 (34.0)	67.1 [58.9–75.7]
VACS 2.0, with albumin, AST and ALT values imputed to the normal value for VACS 2.0 calculations	900 (67.7)	64.8 [57.7–71.8]	1311 (98.6)	65.6 [57.8–72.5]
Complete data for both Dat'AIDS and VACS 1.0	908 (68.3)	NA	1318 (99.1)	NA
Complete data for both Dat'AIDS and VACS 2.0	294 (22.1)	NA	452 (34.0)	NA
Complete data for both Dat'AIDS and VACS 2.0, with albumin, AST and ALAT values imputed to the normal value for VACS 2.0 calculations	900 (67.7)	NA	1311 (98.6)	NA

**TABLE 3:** Description of pre‐specified risk groups of the Dat'AIDS score in the Dat'AIDS score validation study.

Risk groups	Score values	Median age (years)	Median observed score	Number of patients	Observed number of deaths	5‐year prediction^a^	5‐year observed survival probability (95% CI)^b^
Low risk	0–3	71.8	1	340 (25.6%)	17	0.95	0.95 [0.92–0.97]
Moderate risk	4–13	74.1	8	592 (44.5%)	83	0.90	0.85 [0.82–0.89]
High risk	14–19	77.2	16	193 (14.5%)	40	0.80	0.77 [0.71–0.83]
Very high risk	≥20	77.0	25	205 (15.4%)	81	0.53	0.55 [0.48–0.63]

^a^ 5‐year predicted probability of survival using the baseline hazard published in the derivation article [11].

^b^ Kaplan–Meier estimates and 95% confidence interval.

### Discrimination assessment

**FIGURE 1:**
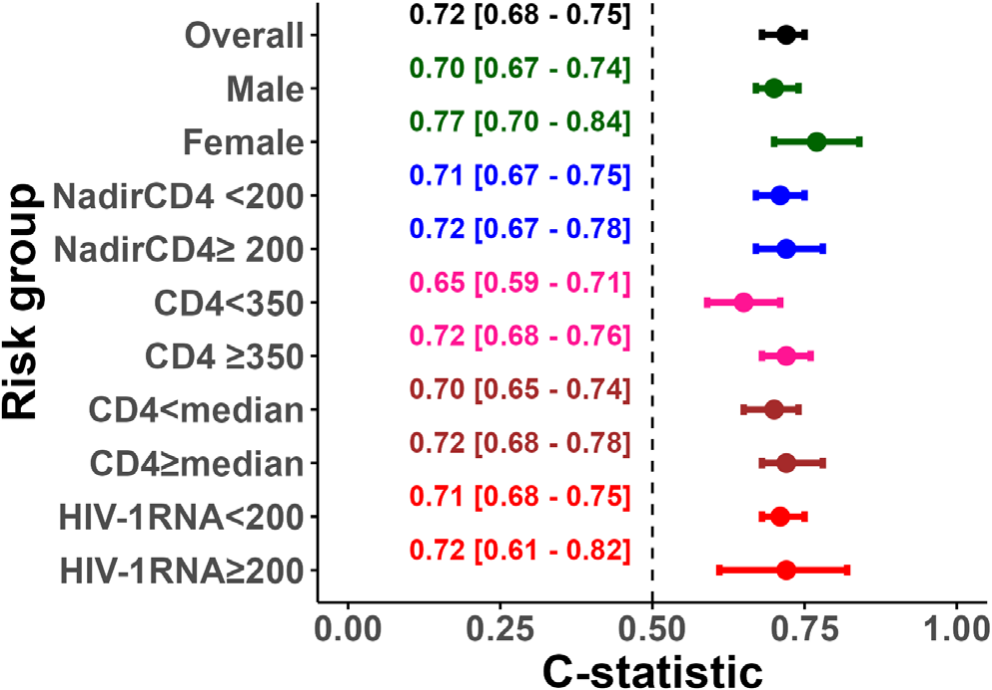
Harrell's C‐statistic for the Dat'AIDS score assessed in the whole dataset and across predefined subgroups. CD4, cells/mm^3^.

The C‐statistic of the Dat'AIDS score in continuous was 0.72 (95% CI [0.68–0.75]), indicating good discrimination. The C‐statistic ranged from 0.65 to 0.77 across subgroups (Figure 1). The only subgroup with a C‐statistic <0.7, specifically 0.65, was the subgroup of patients with a CD4 count <350. Each increase of one risk group in the Dat'AIDS score resulted in a significant 1.5‐to‐3‐fold increase in the HR (Supplementary Table 3).

### Calibration assessment

**FIGURE 2:**
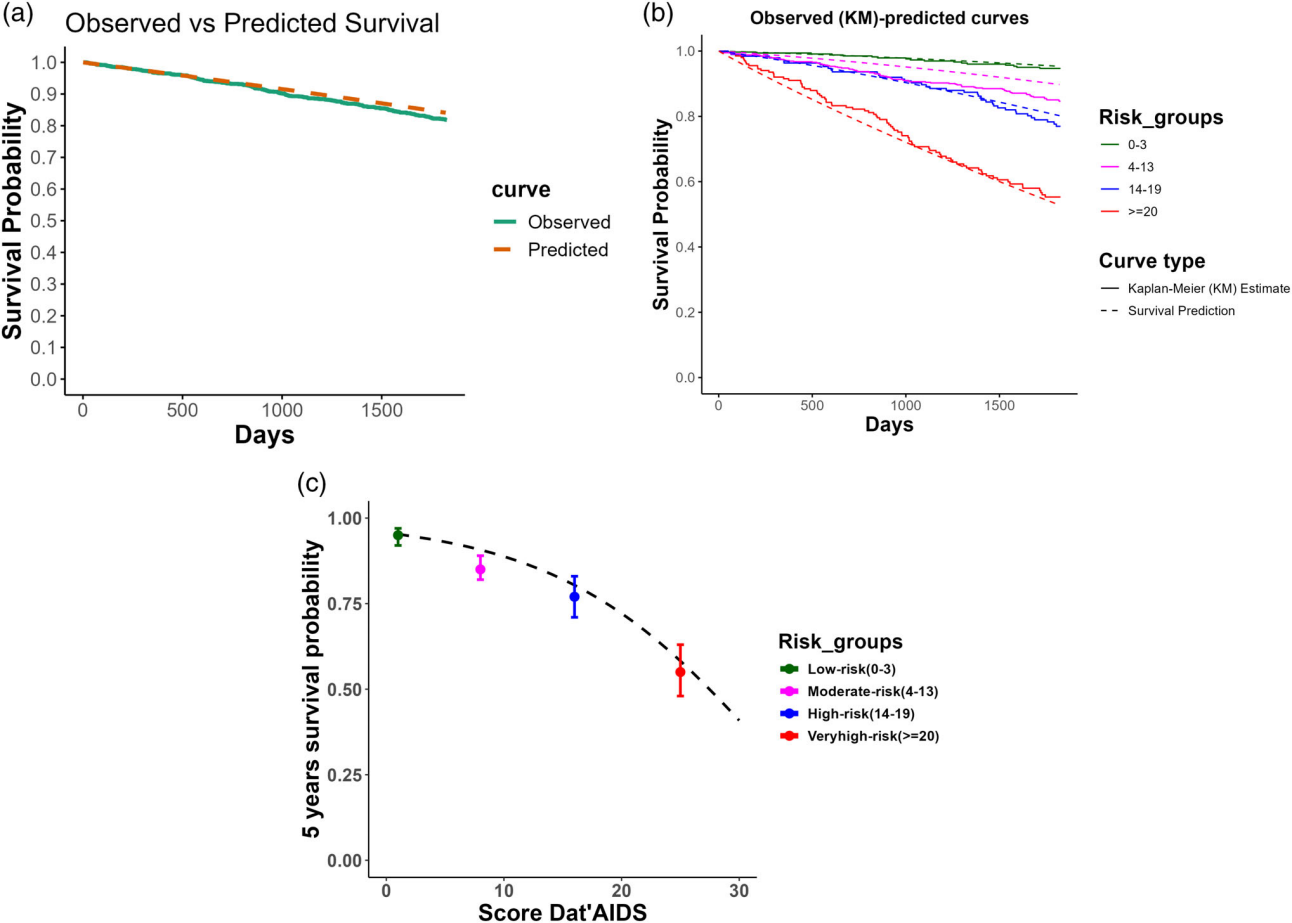
Calibration assessment of the Dat'AIDS score in people living with HIV aged 70 or more. (a) Observed survival (continuous lines, Kaplan–Meier estimates) and predicted survival (dashed lines) probabilities using the score as continuous. Predicted probabilities were calculated using the survival function of the derivation study as previously published [11]. (b) Observed survival (continuous lines, Kaplan–Meier estimates) and predicted survival (dashed lines) probabilities in each pre‐specified risk group. For each risk group, the observed and predicted survival probabilities were similar, suggesting good calibration. Predicted probabilities were calculated using the baseline survival function of the derivation study as previously published [11]. (c) Five‐year observed survival probability and 95% confidence intervals per risk group and predicted survival probability (dashed line) obtained by the Dat'AIDS score. Predicted probabilities were calculated using the baseline survival function of the derivation study. Observed probabilities were centered around the median score of each score group.

The Dat'AIDS score accurately predicted the 5‐year probability of death in continuous and in the low‐, high‐, and very‐high‐risk groups and slightly underestimated the probability of death in the moderate‐risk group (Table 3, Figure 2a–c), with a 5% difference. As a result, the calibration slope was estimated at 0.73 (95% CI 0.62–0.84), and the intercept was 0.07. This difference in calibration remained visible when displaying the observed versus expected survival according to the median observed score in each subgroup (Figure 2b).

### Comparison of the DAT'AIDS score with the VACS 1.0 and 2.0 indices

**FIGURE 3:**
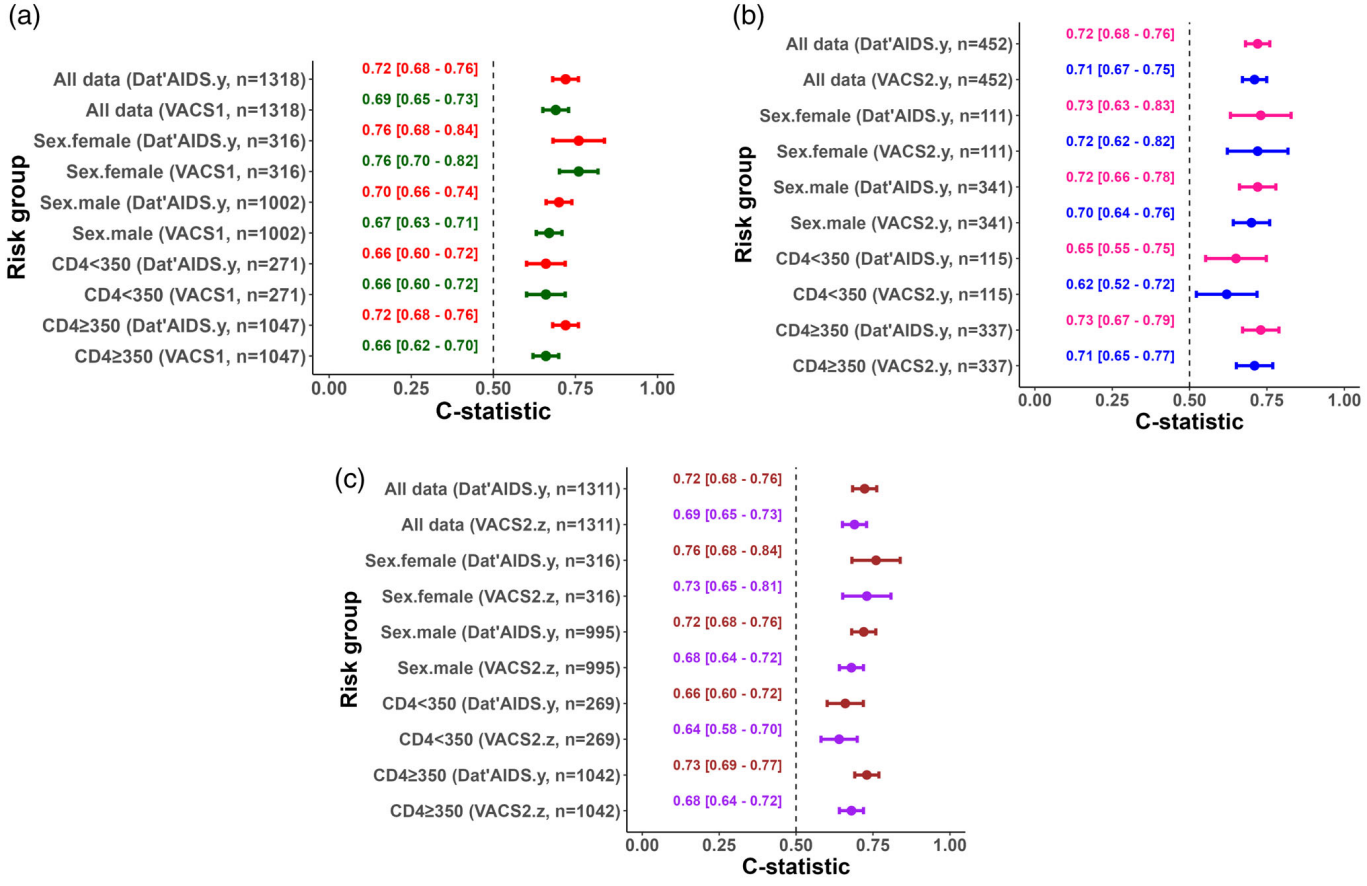
Comparative discrimination capacity assessed by Harrell's C‐statistic and 95% confidence intervals for the Dat'AIDS score and the VACS 1.0 and 2.0 indices in people living with HIV aged 70 or more. Analyses used the common dataset for the Dat'AIDS score and the VACS index 1.0 (a), the common dataset for the Dat'AIDS score and the VACS index 2.0 (VACS2.y) (b), and the common dataset for the Dat'AIDS score and the VACS index 2.0 with albumin, AST and ALT imputed with the normal value (VACS2.z) (c).

The VACS 1.0 and 2.0 indices could be calculated in 99.1% and 34.0% of patients, respectively, increasing to 98.6% for VACS 2.0 when albumin, ALT and AST were also imputed as normal. The C‐statistics for the Dat'AIDS score and VACS 1.0 index were 0.72 (95% CI [0.68–0.76]) and 0.69 (95% CI [0.65–0.73]), respectively, in patients for whom we could simultaneously calculate both scores. Similarly, the C‐statistics for the Dat'AIDS score and the VACS 2.0 index were 0.72 (95% CI [0.68–0.76]) and 0.71 (95% CI [0.67–0.75]), respectively. When albumin, ALT, and AST were additionally imputed as normal, the C‐statistics were 0.72 (95% CI [0.68–0.76]) for the Dat'AIDS score and 0.69 (95% CI [0.65–0.73]) for VACS 2.0 among patients in whom both scores could be calculated. Comparisons of C‐statistics across subgroups among patients for whom both scores could be calculated simultaneously are shown in Figure 3a (Dat'AIDS vs. VACS 1.0), 3b and 3c (Dat'AIDS vs. VACS 2.0).

### Sensitivity analyses

There was no significant difference in the closest CD4 cell count before or after the entry date, and no significant difference in the C‐statistic (Supplementary Figure 3), calibration intercept or calibration slope when restricting the BMI calculation to the closest value measured only in the 12 months before or after entry date (0.72 (95% CI [0.67–0.76]), 0.07 and 0.73 (95% CI [0.62–0.84]), respectively). Similarly, the C‐statistics for the VACS 2.0 index calculated using different methods for data collection and imputation were 0.68 (95% CI [0.62–0.74]), 0.71 (95% CI [0.67–0.75]), and 0.69 (95% CI [0.65–0.73]), respectively (Supplementary Figure 4).

## DISCUSSION

In this study, we assessed the calibration and discriminatory ability of the Dat'AIDS prognostic score for 5‐year overall mortality among people living with HIV aged 70 and older. We also compared the discrimination capacity of the three scores in existence at the time of the study (Dat'AIDS score, VACS 1.0 and 2.0 indices).

In more detail, the discrimination capacity of the Dat'AIDS score in our dataset was good [19, 20, 21] when used as a continuous variable and very similar to that estimated in the external (geographical and temporal) validation study performed in the context of the nationwide SHCS (0.73) [12]. Moreover, it was consistent across all subgroups, except for the subgroup of patients with a CD4 count <350 (C‐statistic 0.65), probably since they are at higher risk of AIDS‐related mortality than from non‐AIDS comorbidities. Discrimination across the four pre‐specified risk groups was good. More specifically, we found that the HR between the low‐ and moderate‐risk groups and the high‐ and very‐high‐risk groups was comparable to those observed in the derivation study [11]. Discrimination between the moderate‐ and high‐risk groups (HR 1.51, 95% CI [1.04–2.20]) was adequate, but lower than that in the derivation study (HR 2.56, 95% CI [1.59–4.12]) [11] and in the external validation in the context of the nationwide SHCS (HR 2.65, 95% CI [1.73–4.07]) [12]. Thus, we can assert that the Dat'AIDS score possesses a good discriminatory capacity, whether employed quantitatively or in pre‐specified groups. This discrimination capacity was as good as in individuals aged over 60 [12]. Regarding calibration, the Dat'AIDS score accurately predicted the risk of five‐year all‐cause mortality in the low‐, high‐, and very‐high‐risk groups and slightly underestimated (5% difference between predicted and observed mortality) the mortality rate of the moderate‐risk group, thus demonstrating a satisfactory level of overall calibration within our study population.

The 5% difference between observed and predicted survival rates in the moderate‐risk group was not explained by a difference in the median Dat'AIDS score between the derivation (median = 8) and the present validation study (median = 8). A possible explanation for this miscalibration could thus be that the increase in age is insufficiently accounted for in the Dat'AIDS score. Indeed, being aged between 64 and 74 years old accounts for only one point in the Dat'AIDS score, whereas being aged 75 or more accounts for 8 points. Perhaps increasing the number of points in the score for patients aged 70 to 74 years could reclassify people living with HIV at higher risk in the high‐risk group, thereby correcting this miscalibration. This will be considered in future updates of the Dat'AIDS score. Finally, sampling variations and selection bias cannot be ruled out. Nevertheless, the 5% miscalibration observed in the moderate‐risk group is unlikely to have a significant clinical impact. In any case, clinicians should be aware that within this subgroup of individuals aged 70 and above, there is a slight underestimation of mortality risk in the moderate‐risk group.

The Dat'AIDS score has been now validated [12] and further validated here in the group of people living with HIV aged 70 or over. Accordingly, it is available for clinical use in all people living with HIV aged 60 or over, as well as for epidemiological and interventional research. It could be useful for clinicians to perform a risk–benefit assessment in the context of clinical decision‐making, with the objective of tailoring preventive interventions. It could also be useful for researchers in order to adjust or stratify analyses. Thus, it should be made available as an online calculator and be implemented in electronic health record (EHR) to facilitate its routine use, as it is for other indices. The Dat'AIDS score has some advantages over other indices, namely (i) it is easy to calculate (though every score are easy to calculate when implemented in EHRs), (ii) it can be used in daily clinical practice and (iii) it contains simple and reliably measured predictors that are all easy to obtain at assessment or in HIV cohorts [11]. While EHR‐based systems can theoretically compute multivariable indices automatically, the required variables are not always routinely available, and the score algorithm is not systematically implemented. Moreover, as the number of required variables increases, so does the probability of incomplete components, leading to imputation strategies whose impact on predictive performance should be documented, as shown here. Therefore, if the Dat'AIDS score demonstrates performances at least comparable to more complex indices, its real‐world applicability could support its use in routine practice. Nevertheless, EHR integration may facilitate automated and repeated calculation of prognostic indices in routine care. Future improvements of the Dat'AIDS score could retain some quantitative predictors as continuous variables; while several were categorized using established clinical cut‐offs, others (e.g., age) were categorized more arbitrarily. Using continuous modelling may improve calibration and may help address the calibration issue discussed above.

The VACS 1.0 index exhibited the lowest discrimination capacity. Conversely, the VACS 2.0 index demonstrated a discrimination capacity similar to that of the Dat'AIDS score in patients for whom both scores could be calculated. However, despite showing a better discrimination capacity than version 1.0, the VACS 2.0 index may be harder to use, particularly when using data from existing observational studies, due to variables not routinely collected in clinical practice, especially the albumin level [12, 29]. When imputing albumin, AST and ALT to their normal level, as previously performed [25], the discrimination capacity of the VACS 2.0 index decreased to that of the one of the VACS 1.0. Moreover, the VACS indices were not specifically designed for the aging people living with HIV population. In patients aged 50 years or older (mostly between 50 and 60 years old), the VACS 1.0 and 2.0 indices had similar C‐statistics, at 0.75 and 0.77 respectively, in the VACS and NA‐ACCORD cohorts [29]. In the context of the SHCS (data not peer‐reviewed) [30], the Dat'AIDS score also showed higher discriminatory capacity measured by the C‐statistic (0.73) compared to the VACS 1.0 index (0.68), in people living with HIV aged 60 or over, but also in the subgroup of people living with HIV aged 65 or over (0.69 vs. 0.62). The discrimination of the VACS 2.0 index was very similar to that of the Dat'AIDS score (0.72 vs. 0.74, respectively), but the discrimination capacity of the Dat'AIDS score was also higher in people living with HIV aged 65 or over (0.72 vs. 0.64). Moreover, whereas the Dat'AIDS score and VACS 1.0 index could both be calculated in about 95% of patients, the VACS 2.0 index could be calculated in only one third of patients, which is very similar to the present study, though the comparison was not performed imputing albumin, AST and ALT.

Our study has several strengths. First, the Dat'AIDS cohort is a large nationwide cohort representative of people living with HIV diagnosed and followed in France, enabling us to assess the validity of the Dat'AIDS score with sufficient statistical power in one of the largest studies including people living with HIV aged ≥70 to date. This is particularly relevant given that external validation studies often reveal a less pronounced discriminatory ability than in the derivation phase [31]. Another strength is that the study period (2014–2019) is very different from that of the derivation study (2008–2013), enabling temporal validation. Also, several HIV centres from around the country joined the cohort since the derivation study, enabling at least partial geographical validation. Finally, systematic information about vital status for patients lost to follow‐up strengthened the assessment of the primary endpoint.

Our study also has some limitations. First, we selected variables that were the closest to the entry date, within 12 months before or after this date, and even later for BMI, and therefore, caution is advised when assessing prognostic indices to be used instantaneously. Nevertheless, sensitivity analyses showed no impact of this evaluation timeframe on the main results. Second, this study was performed in France, in a high‐income context and therefore, results may not be extrapolated to other populations with different incomes, healthcare systems or geographical origins without further study. This caveat notwithstanding, because the variables needed for the calculation of the Dat'AIDS score are available in most clinics, it should be easy to assess the score in other settings and we encourage researchers to assess the performance of the Dat'AIDS score in their specific setting, as well as comparing it to the existing scores (i.e. VACS 1.0 and 2.0 indices, or the recently validated VACS‐CCI index that also includes comorbidities assessed via the Charlson Comorbidity Index [25] but no HIV‐related variables) independently of their derivation cohort. Third, cause of death would have been informative but is not reliably collected in the Dat'AIDS cohort. Fourth, because of the observational nature of the cohort, selection and classification bias cannot be excluded. Fifth, we were not able to assess the calibration of the VACS indices, as the equations required to calculate expected survival probabilities have not been published to our knowledge. Thus, we were not able to compare it to the calibration of the Dat'AIDS score, which should be the purpose of future studies. Finally, we chose a study end‐date of 31 December 2019 in order to exclude the COVID‐19 period. Although we acknowledge that this may have resulted in a loss of statistical power, this choice was made to avoid interactions with COVID‐19 itself, or with the disruption of the healthcare system resulting from the pandemic, which could have made our results more difficult to interpret. This specific period will be the subject of future dedicated studies.

In conclusion, we validated the Dat'AIDS prognostic score in people living with HIV aged 70 years or older, for the prediction of 5‐year mortality risk. The score demonstrated good discrimination and satisfactory calibration in this age group, similar to its performance in the population aged 60 and older. While some minor miscalibration was observed in the moderate‐risk group, it is unlikely to significantly affect the overall clinical utility of the score. While the VACS 1.0 index was as easy to compute as the Dat'AIDS score, it had lower discrimination capacity than the Dat'AIDS score and the VACS index 2.0. Despite similar discrimination capacity, the VACS 2.0 index could be calculated in only one third of patients. When imputing albumin, AST and ALT, the VACS 2.0. index could be calculated in almost every patient but is discriminative capacity then decreased to the level of that of the VACS 1.0. Further studies in diverse populations and settings will help to validate and refine the use of the Dat'AIDS score in different contexts.

## AUTHOR CONTRIBUTIONS

AM, MH and LK designed the study. AM performed data management. AM, MP, LK and MH performed the statistical analyses. AM, LK and MH wrote the first manuscript draft. MH and LK were responsible for the overall supervision of the study. All authors reviewed and approved the study protocol. All authors critically reviewed the manuscript and approved the final version.

## FUNDING INFORMATION

The authors have nothing to report.

## CONFLICT OF INTEREST STATEMENT

The authors have no conflicts of interest to disclose related to the present study.

## Supporting information


**Table S1.** Predictors and their associated points used to compute the Dat'AIDS score.
**Table S2:** Comparison of the variables included in the Dat'AIDS score and in the VACS indices 1.0 and 2.0.
**Table S3:** Model discrimination: hazard ratios across pre‐specified risk groups of the Dat'AIDS score.
**Figure S1:** Flow chart of the study population.
**Figure S2:** Five‐year Kaplan–Meier survival probabilities among each risk group in the validation dataset.
**Figure S3:** Dat'AIDS discrimination assessed by Harrell's C‐statistic and 95% confidence interval calculated using two different methods for BMI data collection in people living with HIV aged 70 or more.
**Figure S2:** Within 12 months before or after inclusion (Dat'AIDS.x) and closest to the inclusion date without time limitations (Dat'AIDS.y).
**Figure S4:** VACS 2.0 index discrimination assessed by Harrell's C‐statistic and 95% confidence interval calculated using two different methods for data collection in people living with HIV aged 70 or more.
**Figure S4:** Closest BMI within 12 months before or after inclusion (VACS2.x), closest BMI to the inclusion date without time limitations (VACS2.y) and closest BMI without time limitation combined with albumin, ALT and AST imputed as normal values when missing (VACS2.z).

## Data Availability

In accordance with the French legislation relating to research involving human beings, and the requirements of the French National Authority for the Protection of Privacy and Personal Data (Commission nationale de l'informatique et des libertés, CNIL), and related to the fact that the dataset concerns HIV‐infected patients, the data underlying this study are restricted. In France, all digital data (including databases, in particular patient data) are protected by the CNIL, the national data protection authority for France. CNIL is an independent French administrative regulatory body whose mission is to ensure that data privacy law is applied to the collection, storage, and use of personal data. As the database of this study was authorized by the CNIL, we cannot make data available without the prior agreement of the CNIL. Interested researchers can send data access requests to the corresponding author.

## APPENDIX A

**Dat'AIDS Study Group:**

C. Chirouze, C. Drobacheff‐Thiébaut, A. Foltzer, K. Bouiller, L. Hustache‐Mathieu, Q. Lepiller, F. Bozon, O Babre, AS. Brunel, P. Muret, E. Chevalier (Besançon), C. Jacomet, H. Laurichesse, O. Lesens, M. Vidal, N. Mrozek, C. Aumeran, O. Baud, V. Corbin, E. Goncalvez, A Mirand, A brebion, C Henquell (Clermont‐Ferrand, France), I. Lamaury, I. Fabre, E. Curlier, R. Ouissa, C. Herrmann‐Storck, B. Tressieres, MC. Receveur, F. Boulard, C. Daniel, C. Clavel, PM. Roger, S. Markowicz, N. Chellum Rungen (Guadeloupe), D. Merrien, P. Perré, T. Guimard, O. Bollangier, S. Leautez, M. Morrier, L. Laine, D. Boucher, P. Point (La Roche sur Yon, France), L. Cotte, F. Ader, A. Becker, A. Boibieux, C. Brochier, F Brunel‐Dalmas, O. Cannesson, P. Chiarello, C. Chidiac, S. Degroodt, T. Ferry, M. Godinot, J.M. Livrozet, D. Makhloufi, P. Miailhes, T. Perpoint, M. Perry, C. Pouderoux, S. Roux, C. Triffault‐Fillit, F. Valour, C. Charre, V. Icard, J.C. Tardy, M.A. Trabaud (Lyon, France), I. Ravaux, A. Ménard, AY. Belkhir, P. Colson, C. Dhiver, A. Madrid, M. Martin‐Degioanni, L. Meddeb, M. Mokhtari, A. Motte, A. Raoux, C. Toméi, H. Tissot‐Dupont (Marseille IHU Méditerranée, France), I. Poizot‐Martin, S. Brégigeon, O. Zaegel‐Faucher, V. Obry‐Roguet, H Laroche, M. Orticoni, M.J. Soavi, E. Ressiot, M.J. Ducassou, I. Jaquet, S. Galie, H. Colson, A.S. Ritleng, A. Ivanova, C. Debreux, C. Lions, T Rojas‐Rojas (Marseille Ste‐Marguerite, France), A. Cabié, S. Abel, J. Bavay, B. Bigeard, O. Cabras, L. Cuzin, R. Dupin de Majoubert, L. Fagour, K. Guitteaud, A. Marquise, F. Najioullah, S. Pierre‐François, J. Pasquier, P. Richard, K. Rome, JM Turmel, C. Varache (Martinique), N. Atoui, M. Bistoquet, E Delaporte, V. Le Moing, A. Makinson, N. Meftah, C. Merle de Boever, B. Montes, A. Montoya Ferrer, E. Tuaillon, J. Reynes (Montpellier, France), B. Lefèvre, E. Jeanmaire, S. Hénard, E. Frentiu, A. Charmillon, A. Legoff, N. Tissot, M. André, L. Boyer, MP. Bouillon, M. Delestan, F. Goehringer, S. Bevilacqua, C. Rabaud, T. May (Nancy, France), F. Raffi, C. Allavena, E. Billaud, C. Biron, B. Bonnet, S. Bouchez, D. Boutoille, C. Brunet‐Cartier, C. Deschanvres, B.J. Gaborit, M. Grégoire, O. Grossi, T. Jovelin, M. Lefebvre, P. Le Turnier, R. Lecomte, P. Morineau, V. Reliquet, S. Sécher, M. Cavellec, E. Paredes, A. Soria, V. Ferré, E. André‐Garnier, A. Rodallec (Nantes, France), P. Pugliese, S. Breaud, C. Ceppi, D. Chirio, E. Cua, P. Dellamonica, E. Demonchy, A. De Monte, J. Durant, C. Etienne, S. Ferrando, R. Garraffo, C. Michelangeli, V. Mondain, A. Naqvi, N. Oran, I. Perbost, M. Carles, C. Klotz, A. Maka, C. Pradier, B. Prouvost‐Keller, K. Risso, V. Rio, E. Rosenthal, I. Touitou, S. Wehrlen‐Pugliese, G. Zouzou (Nice), L. Hocqueloux, C.Mille. T. Prazuck, C. Gubavu, A. Sève, G. Béraud, V. Legros, V. Avettand‐Fènoël (Orléans, France), A. Cheret, C. Goujard, Y. Quertainmont, E. Teicher, N. Lerolle, S. Jaureguiberry, R. Colarino, O. Deradji, A. Castro, A. Barrail‐Tran (Paris APHP Bicètre, France), Y. Yazdanpanah, R. Landman, V. Joly, J. Ghosn, C. Rioux, S. Lariven, A. Gervais, FX. Lescure, S. Matheron, F. Louni, Z. Julia, S. Le GAC C. Charpentier, D. Descamps, G. Peytavin (Paris APHP Bichat, France), C. Duvivier, C. Aguilar, F. Alby‐Laurent, K. Amazzough, G. Benabdelmoumen, P. Bossi, G. Cessot, C. Charlier, P.H. Consigny, K. Jidar, E. Lafont, F. Lanternier, J. Leporrier, O. Lortholary, C. Louisin, J. Lourenco, P. Parize, B. Pilmis, C. Rouzaud, F. Touam (Paris APHP Necker Pasteur, France), MA. Valantin, R. Tubiana, R. Agher, S. Seang, L. Schneider, R. PaLich, C. Blanc, C. Katlama (Paris APHP Pitié Salpétrière, France), F. Bani‐Sadr, M. Hentzien, A. Brunet, D. Lambert, C. Strady, M. Moutel, M. Petithomme‐Nanrocki, V. Greigert, I. Kmiec, H. Marty, V. Brodard (Reims, France), C. Arvieux, P. Tattevin, M. Revest, F. Souala, M. Baldeyrou, S. Patrat‐Delon, J.M. Chapplain, F. Benezit, M. Dupont, M. Poinot, A. Maillard, C. Pronier, F. Lemaitre, C. Morlat, M. Poisson‐Vannier, T. Jovelin, JP. Sinteff (Rennes, France), A. Gagneux‐Brunon, E. Botelho‐Nevers, A. Frésard, V. Ronat, F. Lucht (St‐Etienne, France), D. Rey, P. Fischer, M. Partisani, C. Cheneau, C. Mélounou, C. Bernard‐Henry, E. de Mautort, S. Fafi‐Kremer (Strasbourg), P. Delobel, M. Alvarez, N. Biezunski, A. Debard, C. Delpierre, G. Gaube, P. Lansalot, L. Lelièvre, M. Marcel, G. Martin‐Blondel, M. Piffaut, L. Porte, K. Saune (Toulouse, France), O. Robineau, F. Ajana, E. Aïssi, I. Alcaraz, E. Alidjinou, V. Baclet, L. Bocket, A. Boucher, M. Digumber, T. Huleux, B. Lafon‐Desmurs, A. Meybeck, M. Pradier, M. Tetart, P. Thill, N. Viget, M. Valette (Tourcoing, France).
