# Supplementary material for: Calibration and discrimination ability of the Dat'AIDS score in people living with HIV aged 70 years and older from the Dat'AIDS cohort
Source: HIV Med. 2026 Feb 11;27(5):771–84. doi: 10.1111/hiv.70207 (PMC13140030; doi:10.1111/hiv.70207)
Supplement: Supplementary file 1 — Table S1. Predictors and their associated points used to compute the Dat'AIDS score. Table S2: Comparison of the variables included in the Dat'AIDS score and in the VACS indices 1.0 and 2.0. Table S3: Model discrimination: hazard ratios across pre‐specified risk groups of the Dat'AIDS score. Figure S1: Flow chart of the study population. Figure S2: Five‐year Kaplan–Meier survival probabilities among each risk group in the validation dataset. Figure S3: Dat'AIDS discrimination assessed by Harrell's C‐statistic and 95% confidence interval calculated using two different methods for BMI data collection in people living with HIV aged 70 or more. Figure S2: Within 12 months before or after inclusion (Dat'AIDS.x) and closest to the inclusion date without time limitations (Dat'AIDS.y). Figure S4: VACS 2.0 index discrimination assessed by Harrell's C‐statistic and 95% confidence interval calculated using two different methods for data collection in people living with HIV aged 70 or more. Figure S4: Closest BMI within 12 months before or after inclusion (VACS2.x), closest BMI to the inclusion date without time limitations (VACS2.y) and closest BMI without time limitation combined with albumin, ALT and AST imputed as normal values when missing (VACS2.z). [file HIV-27-771-s001.docx]

**Supplementary Table 1. Predictors and their associated points used to compute the Dat’AIDS score.**

| **Dat’AIDS predictors** | **Points** |
| --- | --- |
| **Age (years)** |  |
| *60-64* | 0 |
| *65-74* | 1 |
| *≥75* | 8 |
| **CD4 (cells/mm^3^)** |  |
| *≥500* | 0 |
| *350-499* | 0 |
| *200-349* | 3 |
| *<200* | 6 |
| **Non-HIV-related cancer** | 6 |
| **Cardiovascular disease^1^** | 8 |
| **eGFR (ml/min/1.73m^2^)** |  |
| *≥60* | 0 |
| *30-59* | 5 |
| *<30* | 16 |
| **Cirrhosis** | 13 |
| **Low BMI^2^** | 10 |
| **Anemia^3^** | 6 |
| BMI: body mass index; eGFR estimated glomerular filtration rate.  ^1^ Cardiovascular disease included history of myocardial infarction, congestive heart failure, cerebrovascular disease  ^2^ Low BMI was defined as a BMI <18.5.  ^3^ Anemia was defined as an haemoglobin level <12g/dL for female and <13g/dL for male. | |

**Supplementary Table 2: Comparison of the variables included in the Dat’AIDS score and in the VACS indices 1.0 and 2.0.**

| **Covariates present in the scores** | **Dat’AIDS score** | **VACS index 1.0** | **VACS index 2.0** |
| --- | --- | --- | --- |
| Age | X | X | X |
| Sex |  |  | X |
| CD4 cell count | X | X | X |
| HIV-1 RNA |  | X | X |
| Non-HIV related cancer | X |  |  |
| Cardiovascular disease | X |  |  |
| eGFR | X | X | X |
| Cirrhosis | X |  |  |
| BMI | X |  | X |
| Hemoglobin level | X | X | X |
| Fib-4 index |  | X | X |
| - Alanine aminotransferase |  | X | X |
| - Aspartate aminotranferase |  | X | X |
| - Platelet count |  | X | X |
| Hepatitis C co-infection |  | X | X |
| White blood cell count |  |  | X |
| Albumin level |  |  | X |

**Supplementary Table 3. Model discrimination: hazard ratios across pre-specified risk groups of the Dat’AIDS score.**

| Risk group | Hazard Ratio | [95%CI] | P value |
| --- | --- | --- | --- |
| Moderate vs. low risk | 3.08 | [1.83 ; 5.19] | <10^−4^ |
| High vs. moderate risk | 1.51 | [1.04 ; 2.20] | 0.03 |
| Very-High vs. High | 2.38 | [1.63 ; 3.48] | <10^−4^ |
| CI: confidence interval | | | |

People living with HIV-1 aged ≥ 70 years

With at least one clinical visit aged ≥ 70 years between June 2014 and December 2017

N= 2,456

Missing variables for the Dat’AIDS score calculation:
- CD4 cell count (n=45)
- Weight (n=23)
- Hemoglobin (n=9)
- Creatinine (n=4)
- Multiple variables (n=35)

Death at the inclusion visit (n=10)

N= 126

Final study population

N= 1,330

**Supplementary Figure 1.** Flow chart of the study population.


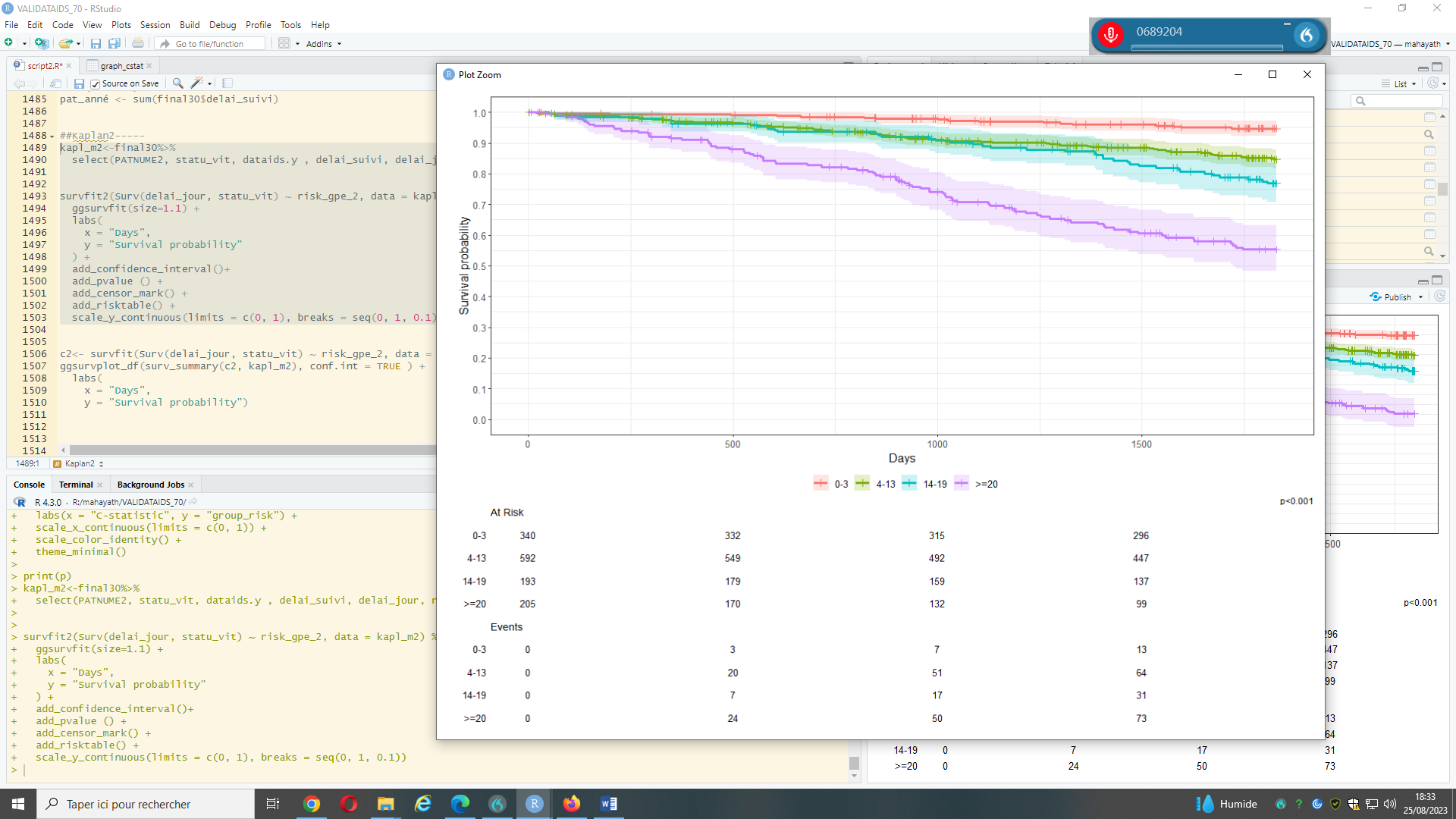


**Supplementary figure 2: Five-year Kaplan-Meier survival probabilities among each risk group in the validation dataset**


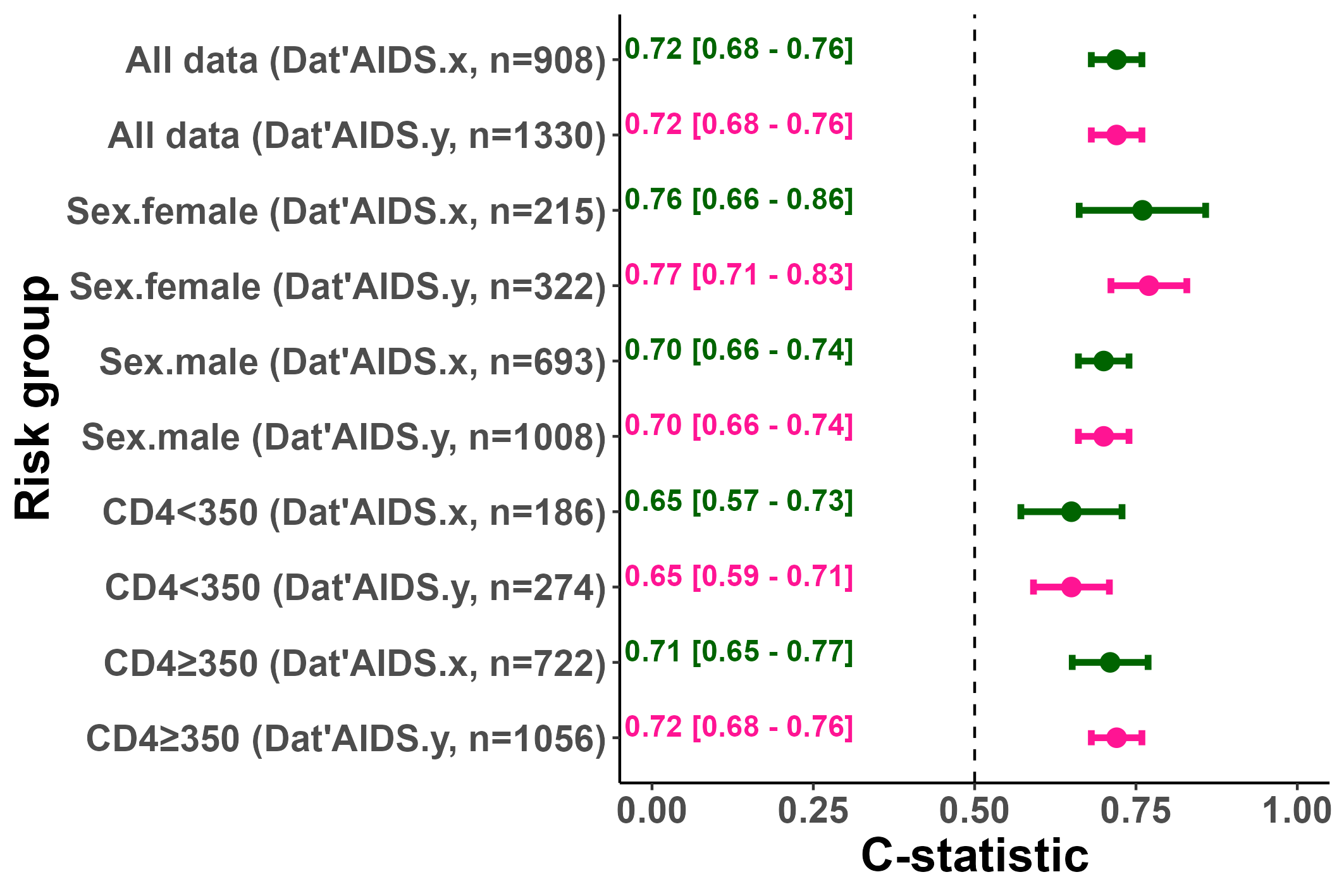


**Supplementary Figure 3: Dat’AIDS discrimination assessed by Harrell’s C statistic and 95% confidence interval calculated using two different methods for BMI data collection in people living with HIV aged 70 or more.**

Supplementary Figure 2 legend: Within 12 months before or after inclusion (Dat’AIDS.x), and closest to the inclusion date without time limitations (Dat’AIDS.y).


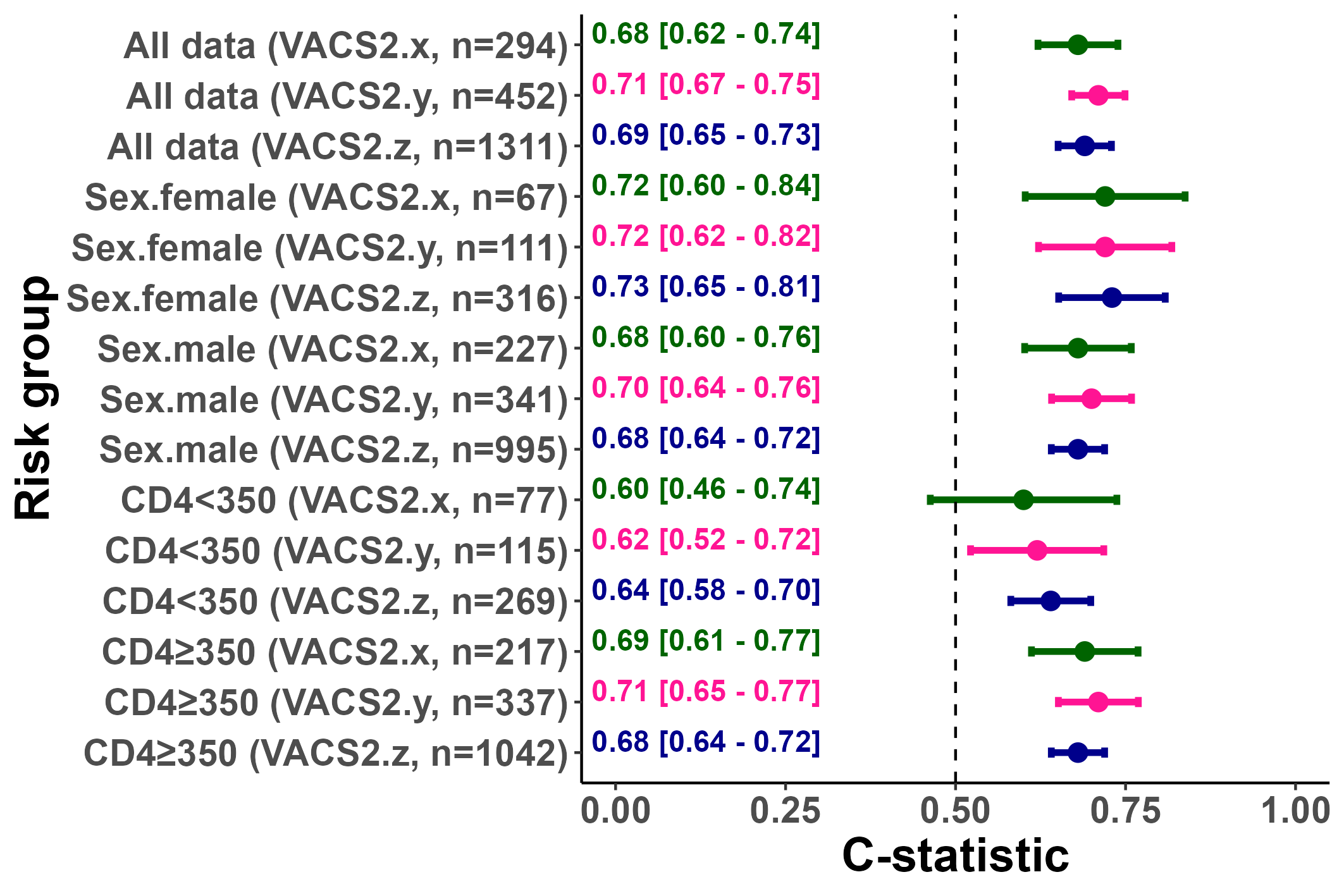


**Supplementary Figure 4. VACS 2.0 index discrimination assessed by Harrell’s C statistic and 95% confidence interval calculated using two different methods for data collection in people living with HIV aged 70 or more**.

***Supplementary Figure 4 legend: closest BMI within 12 months before or after inclusion (VACS2.x), closest BMI to the inclusion date without time limitations (VACS2.y), and closest BMI without time limitation combined with albumin, ALT, and AST imputed as normal values when missing (VACS2.z).***
